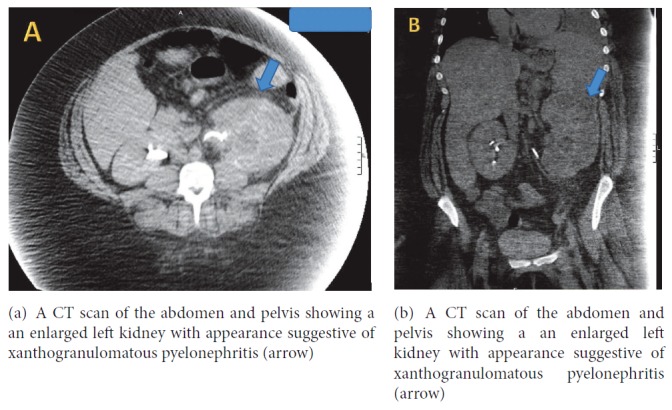# Urinothorax Caused by Xanthogranulomatous Pyelonephritis

**DOI:** 10.1155/2018/7976839

**Published:** 2018-06-14

**Authors:** Waiel Abusnina, Hazim Bukamur, Zeynep Koc, Fauzi Najar, Nancy Munn, Fuad Zeid

**Affiliations:** ^1^Department of Internal Medicine, Joan C. Edwards School of Medicine, Marshall University, Huntington, West Virginia 25701, USA; ^2^Department of Pulmonary Medicine, Joan C. Edwards School of Medicine, Marshall University, Huntington, West Virginia 25701, USA; ^3^Joan C. Edwards School of Medicine, Marshall University, Huntington, West Virginia 25701, USA; ^4^Veterans Affairs Medical Center, Huntington, West Virginia 25704, USA

## Abstract

Xanthogranulomatous pyelonephritis is a rare form of chronic pyelonephritis that generally afflicts middle-aged women with a history of recurrent urinary tract infections. Its pathogenesis generally involves calculus obstructive uropathy and its histopathology is characterized by replacement of the renal parenchyma with lipid filled macrophages. This often manifests as an enlarged, nonfunctioning kidney that may be complicated by abscess or fistula. This case details the first reported case of xanthogranulomatous pyelonephritis complicated by urinothorax, which resolved on follow-up chest X-ray after robot-assisted nephrectomy.

## 1. Introduction

Xanthogranulomatous pyelonephritis (XGP) is an uncommon but distinct form of chronic infective pyelonephritis that typically presents in a woman in her fifth or sixth decade of life with abdominal pain, fever, palpable mass, anorexia, weight loss, hematuria, dysuria, and a persistent urinary tract infection that is resistant to antimicrobial therapy. Diagnosis of XGP is initially made radiographically with histopathological confirmation. Histopathologically, XGP is characterized by replacement of renal parenchyma with granulomatous tissue composed of lipid-laden macrophages [1, 2]. Treatment generally consists of a combination of surgery and antibiotics [3, 4]. Complications of XGP most commonly include abscess or fistula formation [5]. The following case examines the first known reported case of urinothorax as a complication of XGP.

## 2. Case Presentation

A 36-year-old woman with a history of obesity, hypertension, anxiety, and recurrent urinary tract infections (UTI) was admitted to the hospital with history of dyspnea, fever, cough, and abdominal pain for 4 days.

Prior to this admission, the patient presented to urology with recurrent UTIs and was determined to have a left staghorn renal calculus. Recommendations were for the patient to undergo surgical removal of the stone; however the patient refused due to risk of complications associated with her obesity. Up until this admission, the patient had experienced numerous UTIs, most with multidrug resistant bacteria, and had undergone multiple courses of antibiotics.

**Figure 1:**
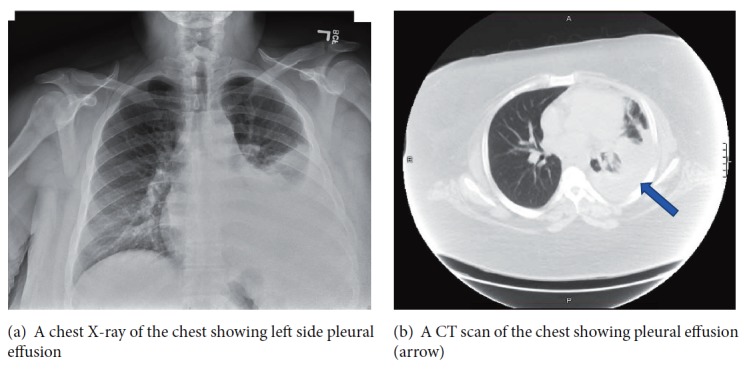


On examination, the patient was dyspneic. The temperature was 100.6 F, the pulse 105 beats per minute, the blood pressure 107/57 mmHg, the respiratory rate 20 per minute, and the oxygen saturation 100% on room air. Complete blood count was significant for a white cell count of 5.8 x 10^3^ per *μ*L. Comprehensive metabolic panel was significant for creatinine 0.76 mg/dL, lactate dehydrogenase 249 IU/L, albumin 3.3 g/dL, and total protein 6.7 g/dL. Chest X-ray (CXR) showed a large left sided pleural effusion with no consolidation (Figures 1(a) and 1(b)). Computed tomography (CT) of the abdomen and pelvis showed an enlarged left kidney with left staghorn calculus in the middle and lower portions of the kidney with an appearance suggestive of xanthogranulomatous pyelonephritis (Figures 2(a) and 2(b)). At this time, a diagnostic thoracentesis was performed yielding lactate dehydrogenase 656 IU/L, total protein 4.5 g/dL, amylase 30 U/L, triglycerides 50 mg/dL,, glucose 105 mg/dL, pH 7.56, and creatinine 0.8 mg/dL. Cultures and cytology of pleural fluid were negative. Pleural fluid was determined to be exudative by Light's criteria as the lactate dehydrogenase was found to be greater than two-thirds the upper limits of our laboratory's normal value [6]. Urine cultures obtained grew extended spectrum beta-lactamase* Escherichia coli* and the patient was started on appropriate antibiotic treatment. Urology service was consulted.

After obtaining a nuclear medicine renal scan with intravenous technetium 99m MAG3 showing significant decrease in left kidney function, the urology consultant recommended and performed a robot-assisted nephrectomy. There was no fistula into the hemithorax identified at the time of nephrectomy. Following this, the patient had a complete resolution of the urinothorax with no evidence of recurrence on follow-up CXR.

## 3. Discussion

Pleural effusion is defined as the accumulation of fluid in the pleural cavity and is classified as either exudative or transudative. Causes are numerous, with differentials based upon the combination of pleural fluid characteristics and clinical presentation. Some causes, such as pelvic tumors, pancreatitis, and carcinoma of the pancreas, are associated with pleural effusion without direct extension to lung tissues. In this case, the source of the pleural effusion was determined to be xanthogranulomatous pyelonephritis (XGP), making it the first reported case of urinothorax (UT) secondary to XGP.

A rare and unusual cause of pleural effusion, UT, describes the phenomenon of urine in the pleural space. Initially described by Corriere et al. in their 1968 study of ureteral obstruction in dogs, UT in human patients has been described as a rare complication of bilateral urinary obstruction or trauma to the urinary tract [7]. The diagnosis of UT requires a diagnostic thoracentesis demonstrating pleural fluid with an average pleural fluid-to-serum creatinine ratio > 1.00 [8, 9]. UT fluid is generally straw-colored and has the distinctive smell of urine. Generally, fluid is characterized as transudative by Light's criteria, that is, containing low protein and low lactate dehydrogenase [6, 10, 11]. In this case of UT, however, the pleural fluid-to-serum creatinine ratio was >1 while the fluid was characterized as exudative due to its elevated lactate dehydrogenase. In a case report by Mora et al., UT can present as an exudative fluid [12]. Additionally, the patient's obstructive uropathy accompanied by recurrent urinary tract infections (UTI) and resolution of the pleural effusion with nephrectomy strongly suggests this to be a case of UT to be secondary to XGP.

UT can be classified as either obstructive or traumatic. The former manifests from a bilateral obstructive uropathy and the latter is most commonly due to iatrogenic injury of the urinary tract [10]. The pathogenesis of transdiaphragmatic extravasation of urine is currently unclear; however many mechanisms, including drainage through the lymphatic system and high pressure through the peritoneal cavity, have been proposed [13].

First described by Schlagenhaufer in 1916, XGP is a rare but serious type of chronic kidney inflammation that can be life-threatening if not treated appropriately [14]. Cases generally present with women in their fifth to sixth decade of life presenting with nonspecific, constitutional symptoms and obstructive uropathy. Radiographically, XGP presents with the “Bear's paw sign,” that is, loss of typical kidney contour combined with dilated calyces and contracted pelvis [15]. Definitive diagnosis requires histopathology [15].

Management of UT requires correction of any underlying potential urinary tract pathology to prevent recurrence and persistence. Currently, no guidelines exist for optimal treatment of XGP but, generally, treatment consists of a combination of broad spectrum antibiotics and nephrectomy [16, 17]. In this case, the patient had a staghorn calculus with recurrent extended spectrum beta-lactamase producing* Escherichia coli* UTIs that lead to development of XGP with subsequent development of urinothorax. Diagnosis was made through chemical analysis of the pleural fluid in addition to imaging. The UT resolved after nephrectomy.

## 4. Conclusion

Urinothorax is a rare diagnosis that is easily overlooked without a high degree of clinical suspicion. This case of urinothorax secondary to xanthogranulomatous pyelonephritis suggests that urinothorax should be included in the differential diagnosis of patients presenting with pleural effusion and recent urinary tract pathologies or nephrolithiasis.

## Figures and Tables

**Figure 2:**